# An Octacalcium Phosphate Forming Cement

**DOI:** 10.6028/jres.115.019

**Published:** 2010-08-01

**Authors:** M. Markovic, L. C. Chow

**Affiliations:** American Dental Association Foundation, Paffenbarger Research Center, National Institute of Standards and Technology, Gaithersburg, MD 20899, U.S.A

**Keywords:** calcium hydroxyapatite, calcium phosphate cement, cement liquids, diametral tensile strength, hardening time, octacalcium phosphate

## Abstract

The osteoconductive and possibly osteoinductive characteristics of OCP increased the interest in preparation of bone graft materials that contain OCP in its composition. Calcium phosphate cements (CPCs) were prepared using a mixture of α-tricalcium phosphate (α-TCP) and dicalcium phosphate anhydrous (DCPA), with α-TCP/DCPA molar ratio of 1/1 and distilled water or 0.5 mol/L phosphate aqueous solution (pH = 6.1 ± 0.1) as the cement liquid. Hardening time was (30 ± 1) min for the CPC mixed with water and (5 ± 1) min for the CPC mixed with phosphate solution. Diametral tensile strength (*DTS*), porosity (*P*), and phase composition (powder x-ray diffraction) were determined after the hardened specimens had been immersed in a physiological-like solution (PLS) for 1 d, 3 d, and 7 d. In CPC specimens prepared with water, calcium hydroxyapatite (HA) was formed and *DTS* and *P* were (9.03 ± 0.48) MPa and (37.05 ± 0.20) vol % after 1 d, respectively, and (9.15 ± 0.45) MPa and (37.24 ± 0.63) vol % after 3 d, respectively. In CPC specimens prepared with phosphate solution OCP and HA were formed and *DTS* and *P* were (4.38 ± 0.49) MPa and (41.44 ± 1.25) vol % after 1 d, respectively,(4.38 ± 0.29) MPa and (42.52 ± 2.15) vol % after 3 d, respectively, and (4.30 ± 0.60) MPa and (41.38 ± 1.65) vol % after 7 d, respectively. For each group *DTS* and *P* did not change with PLS immersion time. *DTS* was significantly higher and *P* was significantly lower for CPCs prepared with water. HA formation slightly increased with immersion time from 40 mass % after 1 d to 50 mass % after 3 d in CPCs prepared with water. OCP + HA formation increased with immersion time from 30 mass % after 1 d to 35 mass % after 3 d and to 45 mass % after 7 d in CPCs prepared with 0.5 mol/L phosphate solution.

## 1. Introduction

Octacalcium phosphate (Ca_8_H_2_(PO_4_)_6_ 5H_2_O; OCP) has been proposed to be a precursor in the formation of apatitic calcium phosphate minerals in bones and teeth [1]. OCP is more soluble and less stable at physiological conditions than calcium hydroxyapatite (Ca_10_(PO_4_)_6_(OH)_2_; HA) [1]. OCP has a layer-type structure composed of alternating hydrated and apatitic layers [2]. The hydrolysis of OCP to HA is thermodynamically favored and it proceeds spontaneously and irreversibly [3,4]. Recent studies demonstrated that synthetic OCP could stimulate osteoblastic cell differentiation *in vitro* [5–8]. *In vivo* studies showed that synthetic OCP was converted to apatitic material in muscle [9] and subcutaneous [10] tissues and at different bony sites [5,10,11]. It was also shown that resorption of OCP was followed by replacement of newly formed bone [12–15]. In addition, OCP coating on metallic implants was reported to promote osteoconductivity [16], osteoblastic cell proliferation [17] and in some cases ectopic osteoinductivity [14,18]. The conversion of OCP to HA itself was suggested to be one of the factors that stimulate osteoblastic cell differentiation [5,8].

The osteoconductive and possibly osteoinductive characteristics of OCP increased the interest in preparation of bone graft materials that contain OCP in its composition. Calcium phosphate cements (CPCs) that are mixtures of two (or more) powdered calcium and/or phosphate containing materials that harden with addition of an aqueous solution are good candidates for formation of OCP. Monma et al., [19] reported that they obtained OCP by reaction of α-tricalcium phosphate (α-Ca_3_(PO_4_)_2_; α-TCP) and dicalcium phosphate dihydrate (CaHPO_4_ 2H_2_O; DCPD) in water. Bermudez et al., [20] reported that OCP was formed in a CPC composed of α-TCP and dicalcium phosphate anhydrous (CaHPO_4_; DCPA) as the solid components and water as the cement liquid, but the hardening time of this CPC was relatively slow (30 min). Sena et al., [21] also suggested formation of OCP in a CPC, consisting of a three component powder mixture (α-TCP + CaCO_3_ + Ca(H_2_PO_4_)_2_) and a phosphate aqueous solution (pH = 7.4), that was used as a pulp filler.

The objective of this study was to prepare fast self-hardening calcium phosphate cement in which OCP is formed. Based on our experience in CPCs with different powder and cement liquid compositions [22–25] and hydrolysis of calcium phosphate compounds [26,27] we hypothesized that the CPC mixture of powdered α-TCP and DCPA, both having appropriate particle sizes could produce formation of OCP in reaction with an aqueous solution.

## 2. Materials and Methods^1^

The α-tricalcium phosphate (α-TCP) with Ca/P molar ratio of 1.50 was prepared by heating a mixture consisting of equimolar amounts of reagent grade calcium carbonate and dicalcium phosphate anhydrous (DCPA) (both from J. T. Baker Chemical Co., Phillipsburg, NJ, U.S.A.) at 1100 °C for 8 h in a furnace (Lindberg, Model 51333, Watertown, WI, U.S.A.) and quenched in air. The α-TCP was ground for 6 min in the planetary ball mill (PM4, Retsch Inc., Newtown, PA, U.S.A.) obtaining 90 mass % of α-TCP particles between 13 μm and 20 μm in diameter with median particle size of 15.8 μm ± 1.2 μm in diameter (mean ± standard deviation; n = 3) (SA-CPR, Shimadzu, Kyoto, Japan). Additional portion of DCPA was ground in ethanol in the planetary ball mill for 24 h. The ground DCPA particles had median diameter of 1.4 μm ± 0.2 μm (n = 3) and 90 mass % of particles had a size distribution between 1.1 μm and 1.6 μm in diameter.

The CPC powdered mixture with a Ca/P molar ratio of 1.33 was prepared by mixing 69.5 mass % of α-TCP and 30.5 mass % of DCPA (molar ratio of α-TCP/DCPA is 1:1). Distilled water or a 0.5 mol/L phosphate solution with pH = 6.1 were used as cement liquids. The phosphate solution was prepared by mixing equal volumes of 0.5 mol/L NaH_2_PO_4_ and 0.5 mol/L Na_2_HPO_4_ solutions. CPC specimens were prepared by placing the powdered mixture on a glass mixing slab, dispensing the liquid on the powder using a micropipette, and mixing the paste using a stainless steel spatula for 30 s. The powder to liquid ratio (P/L) that produced paste with optimal consistency was 3 g/mL.

For hardening time (*HT*) determinations, 400 mg of CPC powder was mixed with 133 μL of cement liquid. The paste was packed into a stainless steel mold with a cylindrical hole of 6 mm in diameter and 3 mm in height. A pressure of 0.7 MPa was applied for 10 s, after which both the top and bottom surfaces of the mold were covered with glass plates and kept in a 100 % humidity box at 37 °C. Because cement hardening occurred very fast when the phosphate solution was the cement liquid, it was desirable to complete the sample preparation procedure within the shortest possible time. In the present study, the sample preparation time starting from delivery of the liquid onto the cement powder to completion of packing the cement paste into the mold was less than 60 s. The *HT* was determined by a large Gilmore needle.

For diametral tensile strength (*DTS*) determination 154.3 mg of the CPC powder was mixed with 51.4 μL of cement liquid. The paste was mixed for 30 s and placed into the cylindrical stainless steel mold as previously described [28]. Briefly, the mold body consists of a cylinder (25.4 mm in diameter and 33.0 mm in height) with a 6 mm diameter hole drilled through the center. Two stainless steel plungers of the same dimensions (6 mm in diameter and 3.20 mm in height) were used. The bottom plunger was dropped into the mold cavity and then the cement paste was placed into the cavity and lightly packed down with a rod. The top plunger was then inserted into the cavity. The mold assembly, with an approximately 6 mm length of each plunger extended out of the mold body, was placed in the constant pressure-loading device with the pressure of 0.7 MPa applied for 4 h [28] and kept in a 100 % humidity box at 37 °C. After 4 h the hardened specimen was removed from the mold and placed in approximately 15 mL of a physiological-like solution (PLS) [29] at 37 °C. The PLS has a composition similar to that of serum in terms of calcium, phosphate, pH, and electrolyte content: *c*(CaCl_2_)= 1.15 mmol/L, *c*(KH_2_PO_4_) = 1.20 mmol/L, *c*(NaCl) = 133 mmol/L, *c*(HEPES) = 50 mmol/L with pH of 7.4 adjusted by addition of KOH. The PLS was replaced with fresh PLS every day. The time periods between sample preparation and *DTS* measurements were 1 d, 3 d and 7 d. Before the *DTS* measurement, the diameter and height of each cylindrical specimen were measured with a micrometer. *DTS* was measured using a Universal Testing Machine (United Calibration Corp., Huntington Beach, CA, U.S.A.) at a loading rate of 10 mm/min.

For density (*d*) determinations, additional *DTS* specimens were dried for 24 h at 37 °C. Diameter (*D*), height (*H*) and mass (*m*) of dried pellet were measured and density (*d*) was determined using the equation *d*=4 *m*/(*D^2^*π *H*). Porosity (*P*), expressed as volume percentage of empty space in the pellet, was calculated from the determined density (*d*) and theoretical density *d_t_*= 3.14 g/cm^3^ of HA [30] using equation *P*= 100 × (1 −*d*/*d_t_*).

The phase composition and morphology of solid phases were determined using powder x-ray diffraction (XRD) (Rigaku 400, Rigaku, Japan) and scanning electron microscopy (SEM) (JEOL SEM 5000, JEOL, Japan), respectively. The amount of non-reacted (residual) α-TCP and DCPA was estimated from reduced intensities of α-TCP and DCPA diffraction lines in CPC samples compared to the initial intensities in α-TCP and DCPA powdered mixture.

A commercially obtained statistical analysis program, Kwikstat (TexaSoft, Cedar Hill, TX, U.S.A.), was used to perform ANOVA (analysis of variance). In this study, the standard deviation was considered as the standard uncertainty for each measured variable.

## 3. Results

The hardening time of the CPC paste with distilled water as a cement liquid was 30 min ± 2 min (n = 6). The *DTS* specimens had diameter in the range from 6.00 mm to 6.03 mm, height in the range from 2.67 mm to 2.83 mm, and mass in the range from 141.3 mg to 150.2 mg. The *DTS*, density and porosity for specimens kept in PLS and determined 1 d or 3 d after preparation are listed in Table 1.

There were no significant (p > 0.05) differences between the values determined at two different times for any of the three measured properties.

**Fig. 1:**
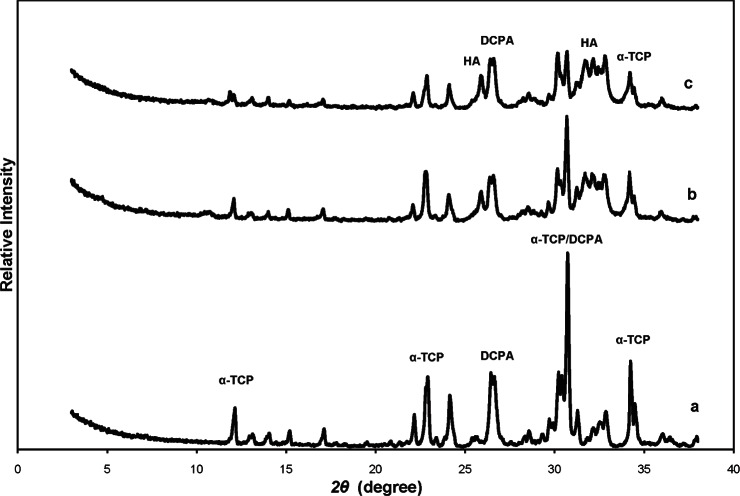
Powder x-ray diffraction (XRD) patterns of (a) initial mixture of calcium phosphate cement (CPC) composed of α-TCP and DCPA powders, (b) CPC product prepared with water as cement liquid and incubated in physiological-like solution (PLS) for 1 d, and (c) CPC product prepared with water as cement liquid and incubated in PLS for 3 d. Diffraction lines characteristic for α-TCP, DCPA and HA are designated.

In Fig. 1 the x-ray diffraction (XRD) patterns of the initial powdered mixture of α-TCP and DCPA (Fig. 1a), and of CPC specimens prepared with distilled water and incubated in PLS for 1 d (Fig. 1b) and 3 d (Fig. 1c) are shown.

The presence of the diffraction lines at 2*θ* of 10.8°, 26.3°, 31.8°, 32.2°, and 32.9° indicated formation of apatitic precipitate (HA) after 1 d and 3 d (Figs. 1b and 1c) The non reacted α-TCP and DCPA were also present (Figs. 1b and 1c). From the intensities of the strongest α-TCP and DCPA diffraction lines in the initial powdered mixture and in the CPC samples after 1d and 3d the estimated contents of non-reacted α-TCP and DCPA after 1 d and 3 d were of about 60 mass % and 50 mass %, respectively. The estimated amount of HA increased from about 40 mass % to about 50 mass %. The absence of the (100) diffraction line at 2*θ* = 4.67° that is typical for OCP indicated that OCP was not formed in these specimens.

**Fig. 2:**
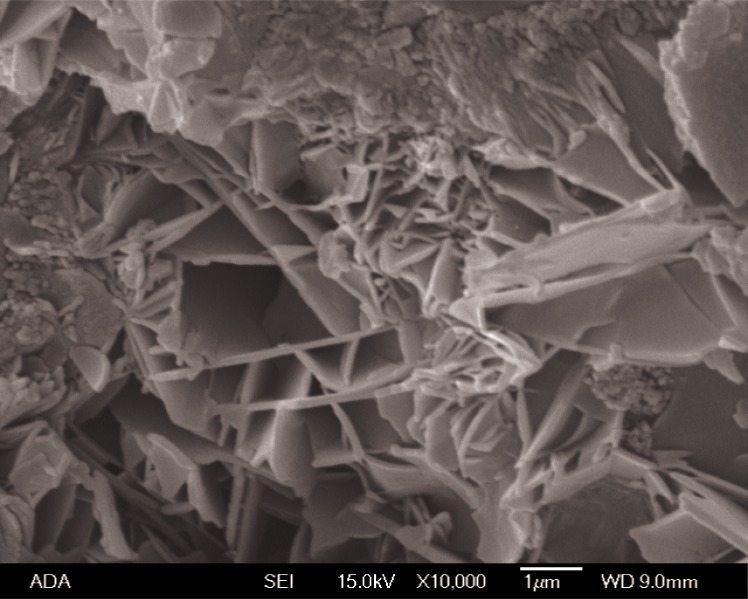
Scanning electron micrograph of reacted calcium phosphate cement product prepared with water as cement liquid and incubated in physiological-like solution for 1 d.

The scanning electron micrograph (Fig. 2) of the sample after 3 d showed that formed HA crystals had plate like morphology with the sizes of 1 μm to 3 μm.

The hardening time of CPC paste using the 0.5 mol/L phosphate solution (pH 6.1) as the cement liquid was 5.0 min ± 0.5 min (n = 6). The *DTS* specimens had diameter in the range from 6.00 mm to 6.02 mm, height in the range from 2.92 mm to 3.34 mm, and mass in the range from 151.7 g to 157.0 g. The *DTS*, density and porosity for specimens incubated in PLS and determined 1 d, 3 d or 7 d after preparation are listed in Table 2. There were no significant (p > 0.05) differences among the values measured at the three time points for *DTS*, *d* or *P*.

**Fig. 3:**
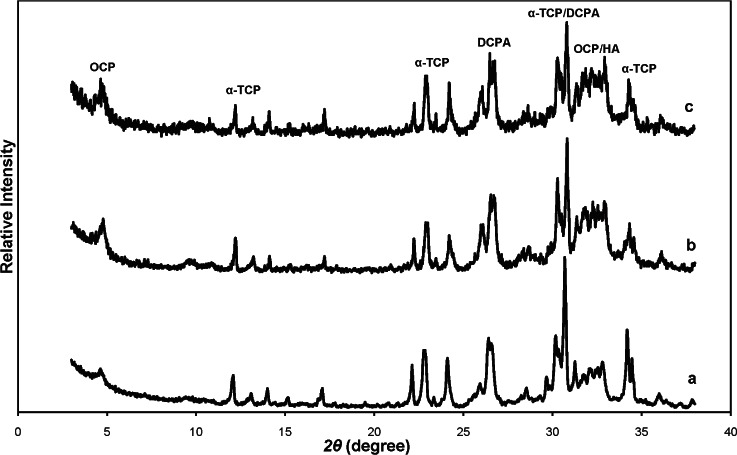
Powder x-ray diffraction (XRD) patterns of calcium phosphate cement products prepared with 0.5 mol/L phosphate solution (pH = 6.1) as cement liquid and incubated in physiological-like solution for (a) 1 d (b) 3 d (c) 7 d. Diffraction lines characteristic for α-TCP, DCPA, OCP and OCP/HA are designated.

The XRD of powdered CPC specimens after 1 d (4 h in water vapor and 20 h in PLS) showed formation of OCP (or OCP-like precipitate with combined OCP and HA layers) and the presence of residual α-TCP and DCPA particles (Fig. 3a). The indication for OCP formation is the presence of the (100) diffraction line at 2*θ*= 4.67° in the XRD pattern. The intensity of this line slightly increased with time for specimens kept in PLS for 1 d (Fig. 3a), 3 d (Fig. 3b) and 7 d (Fig. 3c) indicating slow reaction of α-TCP and DCPA that formed OCP precipitate in the time period from 1 d to 7 d. The estimated amounts of non-reacted α-TCP and DCPA decreased from about 70 mass % after 1 d to about 55 mass % after 7 d. The estimated amount of OCP (or OCP + HA) amount increased from about 30 mass % after 1 d to about 45 mass % after 7d.

**Fig. 4:**
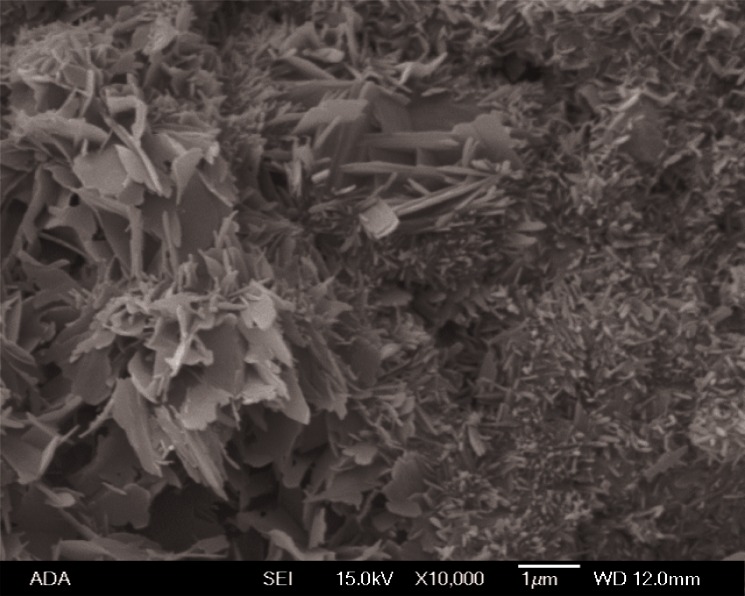
Scanning electron micrograph of calcium phosphate cement product prepared with 0.5 mol/L phosphate solution (pH = 6.1) as cement liquid and incubated in physiological like solution for 1 d.

The scanning electron micrograph of the sample after 1 d (Fig. 4) showed that formed OCP crystals had plate like morphology with the particle sizes of about 0.2 μm to 2 μm.

The CPC samples prepared using the phosphate solution as the cement liquid had significantly (p < 0.05) shorter *HT*, lower *DTS*, smaller density and larger porosity than CPCs samples prepared by using water as the cement liquid.

## 4. Discussion

Previous studies have shown that phosphate solutions significantly shorten the hardening time of several HA-forming CPCs composed of widely different initial ingredients [24, 31, 32]. The faster setting was explained by the more rapid formation of HA crystals under higher phosphate concentration in the cement liquid [22,24]. The present study showed, for the first time, that phosphate solution also accelerated the setting of CPCs that form OCP as a product. Since the OCP formation is likely to be responsible for the cement hardening, this finding suggests that the formation of OCP may also be accelerated by higher phosphate concentrations. The much longer hardening time of 30 min found for the same CPC powdered mixture when distilled water was used as the cement liquid is also in agreement with previous findings for TTCP (tetracalcium phosphate) + DCPA cements that form HA as the product [22, 24, 31, 32]. The longer setting time of about 30 min was also reported by Bermudez et al., [20] who have used distilled water as the liquid and the α-TCP + DCPA powder mixture. Interestingly, as described below, OCP but not HA was found to be the major cement product.

Some of the properties of the CPCs prepared with distilled water as the cement liquid determined in the present study can be compared with the findings of Bermudez et al., [20] in which a cement of the same starting solid components but with different particle sizes and slightly higher P/L ratio were studied. Both the α-TCP and DCPA powders in their system had the median particle sizes of about 5 μm, while in our system the median size of α-TCP was 15.8 μm and that of DCPA was 1.4 μm. The P/L ratio was 3.33 g/mL in theirs and 3.0 g/mL in our system. The measured *HT* of 32 min was almost identical as the *HT* of 30 min measured in the present study. Bermudez et al., [20] reported OCP as the CPC reaction product whereas HA was formed in our CPC, despite the use of water as the cement liquid in both CPCs. It is noteworthy that Bermudez et al., [20] reported that their cement paste had a relatively high initial pH of about 9, which decreased to about pH 8 after 1 d. The higher pH can be attributed to the smaller α-TCP particle size (about 5 μm), leading to a more rapid dissolution. It appears that this higher pH condition was conducive to OCP formation. These findings indicate that even for the same CPC powder mixture of α-TCP + DCPA, the major cement product depended strongly on the solid particle size distributions. Further, for a given cement solid mixture, e.g., α-TCP + DCPA, OCP can form under widely different cement liquid conditions, e.g., water in Bermudez et al. [20] and 0.5 mol/L phosphate solution in the present study. Finally, based on the literature as well data from our preliminary experiments, OCP does not appear to form in any CPC mixture that does not contain α-TCP.

The *DTS* of the OCP-forming CPC reported by Bermudez et al., [20] was about 4.3 MPa, which is nearly identical to the 4.4 MPa found for the OCP-forming cement produced with the phosphate solution as the liquid in the present study. These values are significantly lower than the 9.1 MPa measured for the HA-forming cement produced with the use of water as the cement liquid. The findings suggest that OCP-forming cements are likely to have much lower *DTS* than HA-forming cements, regardless of particle sizes of the cement starting materials.

Although OCP can readily form by the spontaneous hydrolysis of α-TCP at pH of about 6.1 [33,34], our preliminary data indicated that the use α-TCP powder alone (without DCPA) with either distilled water or the phosphate solution did not produce a hardening cement. This suggests that the DCPA was a necessary additional cement component, and its role was to compensate for the differences between α-TCP and OCP in terms of the acid/base content and Ca/P molar ratio. It can be seen from calcium phosphate solubility phase diagrams [35] that DCPA is not more soluble than OCP under neutral and acidic pHs, whereas α-TCP is considerably more soluble than OCP at all pHs below 11 [35]. Thus, it can be concluded that α-TCP and not DCPA is the likely driving force of OCP formation in the α-TCP + DCPA cement.

In the present studies the CPC reaction products were HA or OCP-like precipitate (OCP + HA), depending, respectively, on whether water or a phosphate solution was used as the cement liquid. In either case the large amounts of α-TCP and DCPA remained unreacted; about 55 mass % with phosphate solution after 7 d incubation in PLS, and about 50 mass % with water as the cement liquid after 3 d incubation in PLS. In CPCs composed of TTCP and DCPA, with particle sizes similar to those of α-TCP and DCPA, respectively, used in the present study, HA was the major phase in the product (about 85 mass %) after 1 d with only a small amount (15 mass %) of TTCP left unreacted [23, 25]. TTCP is considerably more soluble than α-TCP for pH < 10 conditions [35], thus it has a greater driving force than does α-TCP for hydrolysis. However, this does not explain why the α-TCP + DCPA → OCP (or HA) reaction was so slow since only about 45 mass % or 50 mass % of the reactants were converted. No XRD data was reported by Bermudez et al. [20], making it difficult to compare the extent of OCP formation in their studies and the present studies. The slow reaction during immersion for up to 7 d in PLS may explain why the porosity and DTS of the cement were unaffected by the incubation.

In our preliminary studies powder mixtures consisting of the same α-TCP and DCPA but with Ca/P molar ratios of 1.25 and 1.40 were prepared. These powdered mixtures when mixed with 0.5 mol/L phosphate solution at P/L of 3.0 g/mL hardened in 5.0 min ± 0.5 min (n = 6) and produced OCP as a product. In other preliminary experiments the α-TCP and DCPA powder mixture with the Ca/P ratios of 1.33 were mixed with 0.5 mol/L phosphate solutions with pHs of 5.4 or 6.6 at P/L of 3 g/mL, and these pastes also hardened in 5.0 min ± 0.5 min (n = 6) and produced OCP precipitates. These preliminary findings indicated that fast hardening OCP forming CPCs with a range of Ca/P ratios and using cement liquids of different pHs can be formulated.

A previous study [11] showed that OCP, in aggregate forms, when implanted into the subperiosteal areas of mice were found to lead to new bone formation more rapidly than HA or Ca-deficient HA. OCP was unique in that fine filaments and granular materials in the newly formed bone matrix were detected around the remnants of OCP particles as short as seven days after implantation. A subsequent study [36] found that when synthetic OCP was implanted into the critical-sized defects in rat calvaria, bone formation was initiated from the margin of the defect and on the implanted OCP away from the margin of the defect. These results support the feasibility of the potential advantages of using bone graft materials that contain OCP as a component. Further *in vitro* and *in vivo* studies are warranted to investigate whether OCP forming CPCs may have greater osteoconductivity than CPCs that form HA, carbonated HA, Ca-deficient HA, or brushite as the major phase.

## 5. Conclusions

The α-TCP and DCPA reaction products were HA or OCP + HA, depending, respectively, on whether water or a phosphate solution was used as the cement liquid. In either case α-TCP and DCPA remained unreacted in the amount of about 50 mass % when water and about 55 mass % when 0.5 mol/L phosphate solution was the cement liquid. The presence of phosphate in cement liquid accelerated the cement hardening from 30 min to 5 min, and induced formation of OCP.

## Figures and Tables

**Table 1:** Diametral Tensile Strength (*DTS*), density (*d*) and porosity (*P*) for CPC samples mixed with distilled water at powder to liquid ratio of 3 g/mL at different reaction times (n = 6 for each group)

Reaction Time	*DTS* (MPa)	*d* (g/cm^3^)	*P* (%)
1 d	9.03 ± 0.48	1.89 ± 0.01	37.05 ± 0.20
3 d	9.15 ± 0.45	1.88 ± 0.02	37.24 ± 0.63

**Table 2:** Diametral Tensile Strength (*DTS*), density (*d*) and porosity (*P*) for CPC samples mixed with 0.5 mol/L Phosphate Solution (pH = 6.1) at powder to liquid ratio of 3 g/mL for different reaction times (n = 6 for each group)

Reaction Time	*DTS* (MPa)	*d* (g/cm^3^)	*P* (%)
1d	4.38 ± 0.49	1.76 ± 0.04	41 44 ± 1 25
3d	4.38 ± 0.29	1.72 ± 0.06	42.52 ± 2.15
7d	4.30 ± 0.60	1.76 ± 0.06	41.38 ± 1.65

## Footnotes

1 Certain commercial materials and equipment are identified in this paper to specify the experimental procedure. In no instance does such identification imply recommendation or endorsement by the National Institute of Standards and Technology, the National Institutes of Health, or the ADA Foundation or that the material or equipment identified is necessarily the best available for the purpose.
